# Autoantibodies Against the Complement Regulator Factor H in the Serum of Patients With Neuromyelitis Optica Spectrum Disorder

**DOI:** 10.3389/fimmu.2021.660382

**Published:** 2021-04-27

**Authors:** Barbara Uzonyi, Zsóka Szabó, Eszter Trojnár, Satu Hyvärinen, Katalin Uray, Helle H. Nielsen, Anna Erdei, T. Sakari Jokiranta, Zoltán Prohászka, Zsolt Illes, Mihály Józsi

**Affiliations:** ^1^ MTA-ELTE Immunology Research Group, Eötvös Loránd Research Network (ELKH), Department of Immunology, ELTE Eötvös Loránd University, Budapest, Hungary; ^2^ Department of Immunology, ELTE Eötvös Loránd University, Budapest, Hungary; ^3^ MTA-ELTE “Lendület” Complement Research Group, Department of Immunology, ELTE Eötvös Loránd University, Budapest, Hungary; ^4^ Department of Internal Medicine and Haematology, Semmelweis University, Budapest, Hungary; ^5^ Research Group for Immunology and Haematology, Semmelweis University-Eötvös Loránd Research Network (Office for Supported Research Groups), Budapest, Hungary; ^6^ Department of Bacteriology and Immunology, Medicum, and Immunobiology Research Program Unit, University of Helsinki and Helsinki University Hospital, University of Helsinki, Helsinki, Finland; ^7^ MTA-ELTE Research Group of Peptide Chemistry, Eötvös Loránd Research Network (ELKH), ELTE Eötvös Loránd University, Budapest, Hungary; ^8^ Department of Neurology, Odense University Hospital and Institute of Molecular Medicine, University of Southern Denmark, Odense, Denmark; ^9^ Department of Neurology, Medical School, University of Pécs, Pécs, Hungary; ^10^ MTA-ELTE Complement Research Group, Eötvös Loránd Research Network (ELKH), Department of Immunology, ELTE Eötvös Loránd University, Budapest, Hungary

**Keywords:** aquaporin (AQP) 4, complement, factor H, neuromyelitis optica spectrum disorder, autoantibody, autoimmunity, inflammation, central nervous system

## Abstract

Neuromyelitis optica spectrum disorder (NMOSD) is an autoimmune inflammatory disease of the central nervous system (CNS), characterized by pathogenic, complement-activating autoantibodies against the main water channel in the CNS, aquaporin 4 (AQP4). NMOSD is frequently associated with additional autoantibodies and antibody-mediated diseases. Because the alternative pathway amplifies complement activation, our aim was to evaluate the presence of autoantibodies against the alternative pathway C3 convertase, its components C3b and factor B, and the complement regulator factor H (FH) in NMOSD. Four out of 45 AQP4-seropositive NMOSD patients (~9%) had FH autoantibodies in serum and none had antibodies to C3b, factor B and C3bBb. The FH autoantibody titers were low in three and high in one of the patients, and the avidity indexes were low. FH-IgG complexes were detected in the purified IgG fractions by Western blot. The autoantibodies bound to FH domains 19-20, and also recognized the homologous FH-related protein 1 (FHR-1), similar to FH autoantibodies associated with atypical hemolytic uremic syndrome (aHUS). However, in contrast to the majority of autoantibody-positive aHUS patients, these four NMOSD patients did not lack FHR-1. Analysis of autoantibody binding to FH19-20 mutants and linear synthetic peptides of the C-terminal FH and FHR-1 domains, as well as reduced FH, revealed differences in the exact binding sites of the autoantibodies. Importantly, all four autoantibodies inhibited C3b binding to FH. In conclusion, our results demonstrate that FH autoantibodies are not uncommon in NMOSD and suggest that generation of antibodies against complement regulating factors among other autoantibodies may contribute to the complement-mediated damage in NMOSD.

## Introduction

Neuromyelitis optica spectrum disorder (NMOSD) is a rare inflammatory disease of the central nervous system (CNS) with a prevalence of 0.7-10/100,000 worldwide (1) and is characterized by pathogenic complement-activating autoantibodies against aquaporin 4 (AQP4), the main water channel of the CNS (2, 3). Most commonly, relapsing and often bilateral optic neuritis, longitudinally extensive transverse myelitis, and brainstem symptoms characterize NMOSD (4–7).

Complement is an essential effector system of the humoral arm of innate immunity (8). The complement system can be activated *via* three main pathways, the classical, the lectin and the alternative pathways. It provides a first-line defense against infections, participates in the clearance of immune complexes and cellular waste, and influences adaptive immune responses (8–10). Complement gene mutations and polymorphisms that result in altered protein function and thus excessive activation, inappropriate regulation or failure in proper targeting of complement attack may lead to pathogenic complement activation, which by causing inflammation and tissue damage is implicated in the pathogenesis of several diseases (11). In addition to genetic alterations, autoantibodies to complement proteins can cause or contribute to diseases *via* binding to their target, which in turn may impair the function of the respective proteins and result in pathological complement activation (12–14).

Complement has also been implicated in the pathogenesis of NMOSD. CNS lesions are characterized by deposition of complement proteins along with IgG, IgM, and loss of astrocytic AQP4 (15–17). Patients with NMOSD have higher levels of complement activation products in the blood, and the three complement pathways are functionally abnormal even during the remission period (18–20). Autoantibodies to complement C1q were described in NMOSD patients (19). Autoantibodies can activate complement *via* the classical pathway when bound to their target proteins, which was recently described for AQP4-autoantibodies, as well (5, 21). AQP4 autoantibodies, also known as NMO-IgG, mainly belong to the IgG1 subclass, and astrocytes transfected by AQP4 are susceptible to cell death by IgG and IgM AQP4-antibodies in the presence of complement (22, 23). The pathogenic role of NMO-IgG in the presence of human complement is also supported by the results of passive transfer experiments (5, 21) and *in vivo* disease models, where complement inhibition was proven to be beneficial (24). Moreover, clinical experience also sustains the role of complement activation in disease pathogenesis, since treatment with the monoclonal anti-C5 antibody eculizumab reduced attack frequency, and stabilized or improved neurological disability of patients with NMOSD (25, 26). Thus, therapeutic complement inhibition is a promising strategy in the treatment of NMOSD (24, 27), and eculizumab has been approved for treating AQP4-IgG positive NMOSD.

Of the three major pathways of complement, the alternative pathway is particularly powerful because *via* the so-called amplification loop it can enhance complement activation initiated by any complement pathway (8, 28, 29). This is based on the generation of C3b and formation of the C3bBb alternative pathway C3 convertase (30). Therefore, proper regulation of the alternative pathway is essential in maintaining homeostasis. Antibodies to components of the alternative pathway may result in a wide spectrum of diseases. Antibodies to the C3 convertase, i.e. C3 nephritic factors (C3NeFs), may stabilize the convertase resulting in increased complement activation, which has been associated with C3 glomerulopathies (13, 14, 31). Autoantibodies to factor H (FH), the major soluble regulatory protein of the alternative complement pathway, are described in kidney diseases such as atypical hemolytic uremic syndrome (aHUS) and dense deposit disease, and are thought to cause pathogenic complement activation by blocking functional domains of this complement inhibitor protein (14, 32–34).

FH is a 155-kDa serum glycoprotein that upon binding to C3b inhibits the activation of the alternative pathway and the amplification loop. FH acts as a cofactor for the serum protease factor I in the enzymatic inactivation of C3b (cofactor activity), prevents assembly of the C3bBb convertase and accelerates its disassembly if already formed (decay accelerating activity) (35, 36). FH is composed of 20 homologous domains termed short consensus repeats (SCRs), of which SCR1-4 mediate the cofactor and decay accelerating activities of the protein, and SCR19-20 function as recognition domains for deposited C3b/C3d in the context of host surface glycans (37, 38). The physiological function of FH is critical for proper complement regulation. Altered FH activity caused by genetic changes and autoantibodies are associated with several inflammatory and autoimmune pathologies, such as age-related macular degeneration, C3 glomerulopathies and aHUS (36, 39).

Overactivation of the complement system was proven to be present in NMOSD; however, it is unclear what steps lead to complement activation in the pathogenesis of this disease. We hypothesized that autoantibodies against complement proteins may contribute to abnormal complement activation in NMOSD. Therefore, the aim of this study was to evaluate the presence of antibodies against the alternative pathway convertase C3bBb, its components C3b and factor B (FB) as well as the regulator protein FH in the serum of patients with AQP4-seropositive NMOSD.

## Materials and Methods

### Patients

Serum or EDTA-plasma samples of NMOSD patients were collected after informed consent in accordance with the Declaration of Helsinki. The study was approved by the National Ethical Committee (3893.316-12464/KK4/2010 and 42341-2/2013/EKU). Forty-five patients having NMOSD were included in this study and they all were seropositive for anti-AQP4 antibody determined by a commercially available cell-based assay (Euroimmune, Lübeck, Germany). The patients did not present other autoimmune diseases, cancer or infections. They were negative for antinuclear antibodies, except for patient #113; this was a single abnormality, no specific antigen was identified, and no other systemic autoantibodies (anti-dsDNA, anti-SSA, anti-SSB) were detected. Characteristics of the FH autoantibody-positive patients are summarized in **Table 1**.

**Table 1:** Characteristics of NMOSD patients with FH autoantibodies.

	Age at onset (y)	Diagnosis	AQP4-Ab
NMO64	23	relapsing ON and LETM	+
NMO84	41	relapsing ON and LETM	+
NMO113	54	relapsing ON and LETM	+
NMO210	64	LETM	+

All four patients positive for FH autoantibodies are female and have NMOSD for >5 years. All patients have anti-AQP4 antibodies (AQP4-Ab) in their serum as determined by a cell-based assay. ON: optic neuritis, LETM: longitudinally extensive transverse myelitis.

### Proteins, Sera and Antibodies

Purified human FH, FB, C3b, factor D, C1q, goat anti-human C1q antibody (Ab) and goat anti-human FH antibody (Ab) were purchased from Merck (Budapest, Hungary). Human serum albumin (HSA), bovine serum albumin (BSA), alpha1-antitrypsin, HRP-conjugated anti-human IgG, HRP-conjugated anti-human IgA, HRP-conjugated anti-human IgM, and monoclonal antibodies (mAbs) specific for IgG1, IgG2, IgG3, IgG4, Ig kappa and Ig lambda were purchased from Sigma-Aldrich (Budapest, Hungary). HRP-conjugated goat anti-mouse Ig and HRP-conjugated rabbit anti-goat Ig were purchased from DakoCytomation (Hamburg, Germany). HRP-conjugated goat anti-human C3 was from MP Biomedicals (Solon, OH). The anti-FH mAb A254 was purchased from Quidel (Biomedica, Budapest, Hungary), and the mAb C18 (40) was from Alexis Biochemicals (Lörrach, Germany). The anti-FH mAb IXF9 was described earlier (41).

Codon-optimized sequences of FHR-1, FHR-4B, FH SCRs 1-4, FH SCRs 8-14, FH SCRs 15-20 were synthesized (GenScript, Piscataway, NJ) and cloned into the pBSV-8His baculovirus expression vector, expressed in *Spodoptera frugiperda* Sf9 cells and purified by nickel affinity chromatography as described previously (42, 43). FH SCRs 19-20 and mutant 19-20 fragments were expressed in *E. coli* (44).

### Microtiter Plate Assays

Microtiter plate wells were coated with 5 µg/ml FH, FB, C3b, or HSA as negative control antigen, for 1 h at 20°C. To measure autoantibody binding to solid-phase C3bBb convertase, the convertase was built up in microtiter plate wells as previously described (45). After blocking with 5% BSA and 0.1% Tween-20 in phosphate buffered saline (BSA-PBS), patients’ serum samples diluted 1:50 in Dulbecco’s PBS (DPBS; Lonza, Budapest, Hungary) were added for 1 h. Bound IgG was detected by incubating the wells with HRP-conjugated anti-human IgG for 1 h. Color reaction was developed with TMB (Kem-En-Tec Diagnostics, Taastrup, Denmark) and absorbance was measured at 450 nm. Antibody positivity was determined based on the reactivity with the specific antigen and the negative control protein; those having an OD value ≥ the double of that of the control protein were considered positive. The identified samples were analyzed in additional assays (see below) to confirm autoantibody positivity and characterize specific binding sites and potential functional effects of the autoantibodies.

To detect IgM and IgA autoantibodies, samples were preincubated with Protein G-agarose beads (Sigma-Aldrich) to deplete IgG, and these IgG-depleted samples were added to wells coated with 5 µg/ml FH. The presence of IgM and IgA autoantibodies was detected as described above, except for using the corresponding HRP-conjugated detection antibodies instead of anti-human IgG. In some assays, prior to immobilization FH was treated with 10 mM Tris(2-carboxyethyl)phosphine (TCEP; Sigma-Aldrich) to generate reduced FH (46). To this end, 20 mM TCEP dissolved in 0.4 M Tris pH 7.4 was mixed 1:1 with 1 mg/ml FH and incubated for 30 min at 20°C. FH was then diluted and immobilized on microplate wells, and used for autoantibody binding assay as described above. Autoantibodies against C1q were analyzed as described previously (45). Briefly, microtiter plates were coated with 2 µg/ml C1q and, as negative control antigens, HSA and α1-antitrypsin. After blocking and washing, serum samples diluted 1:50 in DPBS containing 1 M NaCl were added. Autoantibody binding was detected with HRP-conjugated anti-human IgG diluted in DPBS containing 1 M NaCl.

To map the antibody binding sites within FH, recombinant FH fragments were immobilized and autoantibody binding was detected as described above. For the characterization of IgG isotypes, FH and HSA were immobilized and, after blocking with BSA-PBS, the plates were incubated with patients’ samples. For the detection, mAbs specific for IgG1, IgG2, IgG3, IgG4, Ig kappa and Ig lambda, followed by HRP-conjugated goat anti-mouse Ig, were used. To analyze the effect of anti-FH mAbs, the wells were incubated with the anti-FH mAbs prior to the addition of patients’ sera as described (33). To determine the avidity of the FH autoantibodies, NaSCN as a chaotropic salt was used as described (47, 48). Briefly, after incubation of the wells with the patients’ sera, 0.5 M NaSCN was added for 15 min at 20°C and, after washing, the bound IgG was detected with HRP-conjugated anti-human IgG. Titers of the samples were calculated based on a standard curve and avidity index was calculated as the ratio of bound antibodies in the presence and absence of NaSCN. To calculate the avidity profile, various concentrations of NaSCN were used. To measure the inhibitory effect of autoantibodies on C3b binding, wells were coated with FH19-20 at 5 µg/ml. After blocking with BSA-PBS, the wells were incubated with 500 µg/ml purified IgG, then 2 µg/ml C3b was added. C3b-binding was detected by HRP-conjugated anti-human C3.

### Western Blot

The presence of native FHR-1 was analyzed by Western blotting. To this end, 0.4 µl patient serum diluted in non-reducing sample buffer was run on 10% SDS-PAGE. Proteins were blotted onto a nitrocellulose membrane, and after blocking, the membrane was incubated with the anti-FH mAb C18, which recognizes both FH and FHR-1 (40), followed by HRP-conjugated goat anti-mouse Ig. The blot was developed using the ECL detection kit (Merck).

### IgG Isolation and Analysis

10 µl serum diluted in DPBS was incubated with protein G beads (Life Technologies, Budapest, Hungary) for 2 h at 20°C. After washing, the bound IgG fraction was eluted with non-reducing sample buffer and analyzed for the presence of FH and FHR-1 by SDS-PAGE and Western blotting using the anti-FH mAb C18 followed by HRP-conjugated goat anti-mouse Ig for detection.

For epitope analysis of FH autoantibodies immobilized peptides were used. To this end, acetylated linear 15-mer peptides overlapping in 10 amino acids, and covering the FH SCRs 19-20 (amino acids 1107-1231) as well as their S1191L and V1197A modified peptides corresponding to the homologous FHR-1 sequence were designed. The peptides were prepared in duplicates on functionalized hydroxypropylmethacrylate non-cleavable gears of a nominal capacity of 66 nmol (Mimotopes, Clayton Victoria, Australia) by solid phase Fmoc/tBu peptide synthesis according to Geysen’s method (49), as described earlier (50), with slight modifications. Briefly, the Fmoc protecting groups were removed by 2 v/v% piperidine/2 v/v% 1,8-diazabicyclo[5.4.0]undec-7-ene in N,N-dimethylformamide, the Fmoc-protected amino acid derivatives were coupled by N,N′-diisopropylcarbodiimide/1-hydroxybenzotriazole in N,N-dimethylformamide using ~200 eq reagents. After building up the peptide chains, the N-terminal α-amino group was acetylated and the side chain protecting groups were cleaved with TFA/thioanisole/phenol/water/EDT 82.5:5:5:5:2.5 (v/v/v/v/v).) The peptides remained covalently attached to the gears and were used in linear epitope mapping of the anti-FH autoantibodies.

Autoantibody binding to the synthetic peptides was detected using a modified ELISA described earlier (51). After blocking the non-specific binding sites with 0.5% gelatin in PBS, the gears were incubated with 150 μl of 1:600 diluted sera in PBS/0.5% gelatin/0.05% Tween-20 for 1 h at 20°C. Autoantibody binding was detected using HRP-conjugated rabbit anti-human IgG (DakoCytomation) and TMB detection system. Gears were used repeatedly after thorough cleaning by sonication in PBS containing 1% SDS and 0.1% 2-mercaptoethanol. The ODs were normalized by the following formula: OD_sample_/OD_min_, where OD_sample_ is the mean of duplicate OD values of the test samples and OD_min_ represents the mean binding to the negative control HSP 480-489 peptide, chosen based on our previous study (50). Data were further normalized to OD obtained with sera of healthy controls.

## Results

### Identification of FH Autoantibodies in NMOSD Sera

**Figure 1:**
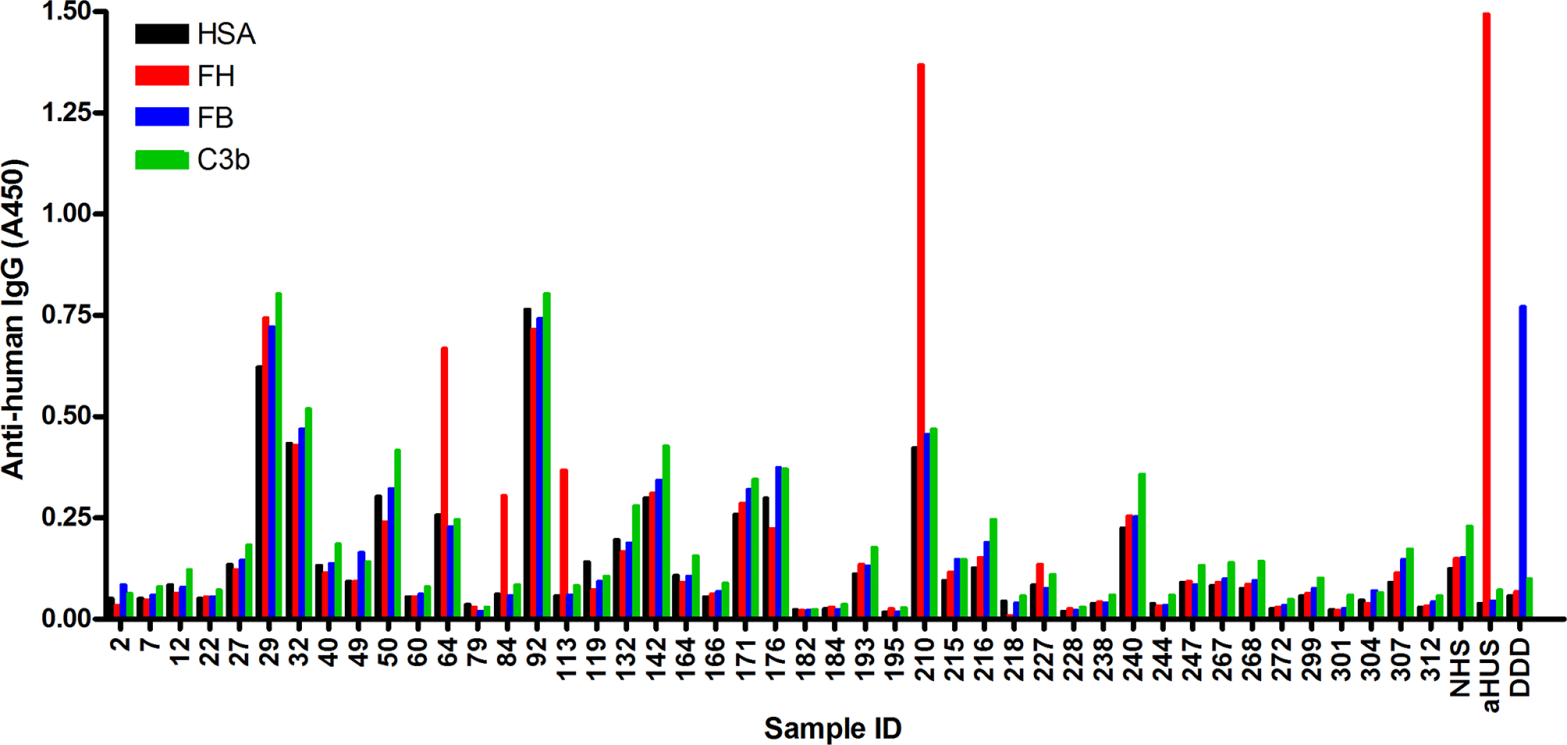
Screening of NMOSD sera for autoantibodies by ELISA. Microplate wells were coated with human serum albumin (HSA), FH, FB and C3b, and after blocking, incubated with sera of 45 NMOSD patients and controls (all serum samples diluted 1:50 in PBS). Binding of autoantibodies to these antigens was detected using HRP-conjugated anti-human IgG. Serum sample of a patient with atypical hemolytic uremic syndrome (aHUS) and of a patient with dense deposit disease (DDD), positive for FH and FB autoantibodies, respectively, were used as positive controls. NHS: normal human serum. Data are means of two measurements. Some samples showed reactivity or high background with all four antigens, and these were considered autoantibody negative. The four samples #64, #84, #113 and #210 showing clearly stronger reactivity with FH compared to reactivity with HSA, FB and C3b, were considered autoantibody positive.

In order to identify autoantibodies to complement proteins in sera of NMOSD patients, ELISA was performed using immobilized FH, C3b and FB, as well as solid-phase C3bBb convertase. Of the 45 samples analyzed, four were positive for FH autoantibodies (i.e., ~ 9%), and specific autoantibodies to C3b, FB or C3bBb were not detected in any of the samples (**Figure 1** **and data not shown**). Some samples showed high background binding; these were considered negative for the autoantibodies if binding to all antigens, including HSA, was similar. In addition, we depleted IgGs from these autoantibody positive samples to facilitate the detection of IgM and IgA isotype FH autoantibodies, if present. There was no specific signal detected for these samples, except for the IgG-depleted serum of patient #210, where slight IgA positivity was observed (**Supplementary Figure 1**). We also tested the presence of autoantibodies to C1q; a few but none of the FH autoantibody positive NMOSD serum samples were positive for C1q autoantibodies (**Supplementary Figure 2**), confirming previous report (19).

**Figure 2:**
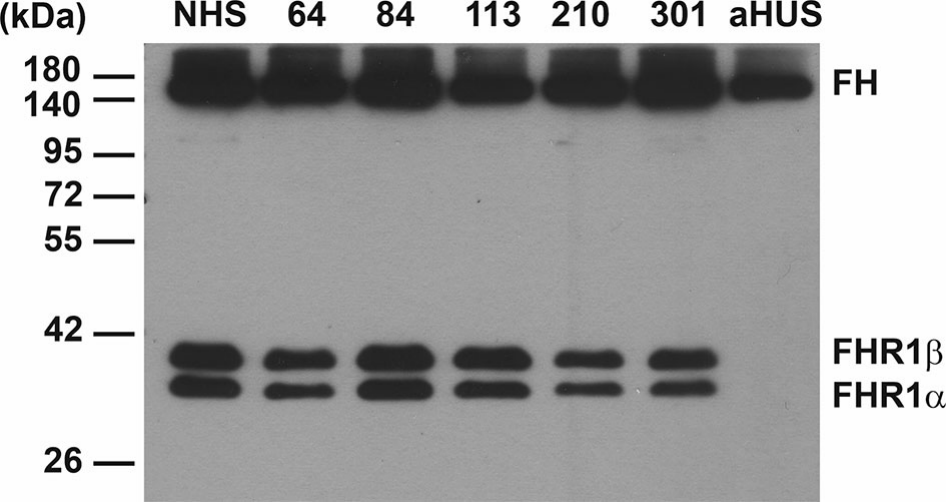
Western blot analysis of NMOSD sera for FHR-1 protein. 0.4 µl of serum samples were run on 10% SDS-PAGE and the blot was developed using the anti-FH mAb C18. The two isoforms of factor H-related protein 1 (FHR-1) are seen in the 4 FH autoantibody positive NMOSD patients (64, 84, 113, 210), in an NMOSD patient (301) negative for FH autoantibody, and in a healthy control sample (NHS). FHR-1 is missing in a FH autoantibody positive aHUS patient, used as a control sample, as typical for ~90% of aHUS patients with FH autoantibodies. The blot is representative of two experiments.

Because FH autoantibodies are strongly associated with the deletion of the *CFHR1* gene in aHUS (52, 53), we investigated the sera of the above four patients for the presence of FHR-1 protein, since DNA samples were not available. The patients did not receive plasma treatment, thus exogenous origin of FHR-1 could be excluded. All four patients had both FHR-1 isoforms in their serum as detected by Western blot analysis (**Figure 2**).

**Figure 3:**
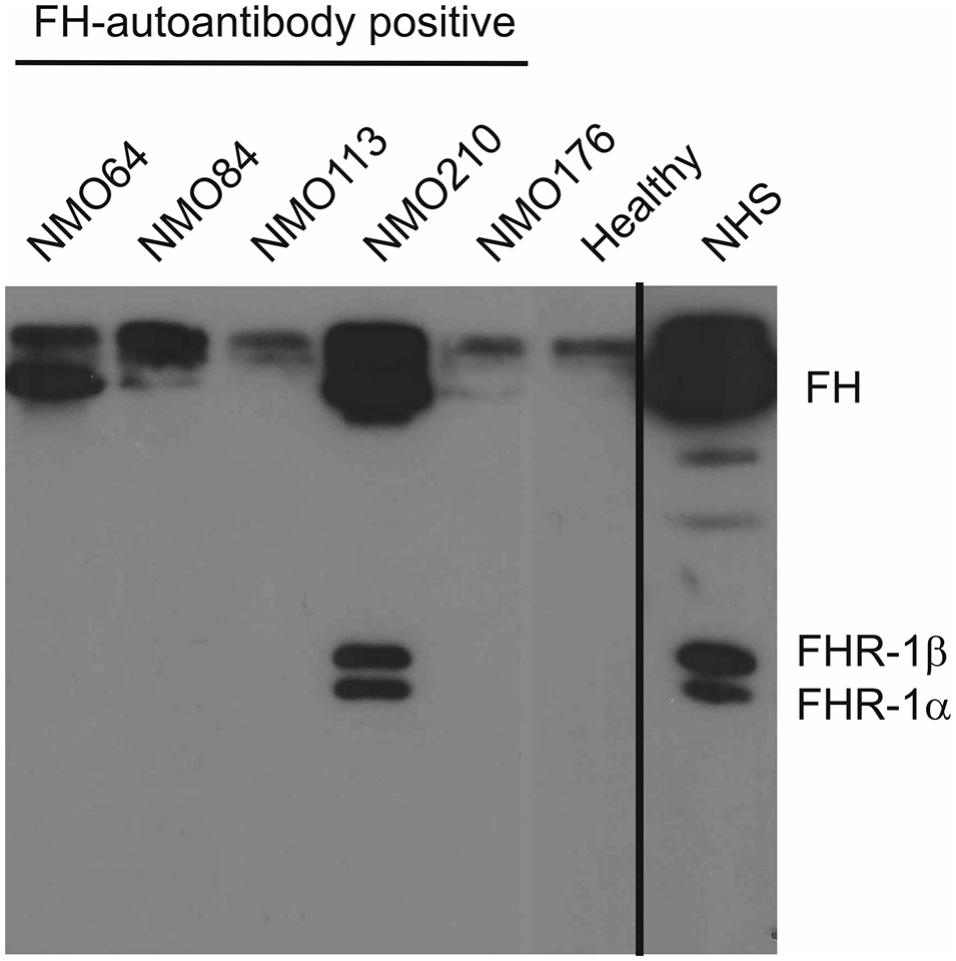
Detection of FH–autoantibody complexes. Western blot analysis of the IgG fractions for FH/FHR-1 – IgG complexes. 10 µl of serum samples were incubated with Protein G beads. The bound proteins, eluted with SDS-sample buffer, were run on 10% SDS-PAGE, transferred to nitrocellulose membrane, and the blot was developed with monoclonal anti-FH recognizing also FHR-1 (mAb C18). FH is detected in the FH-autoantibody positive NMOSD patients (NMO64, NMO84, NMO210), but not in the FH-autoantibody negative (NMO176) or healthy control sample. In addition, FHR-1 is also seen in the NMOSD sample with the highest autoantibody titer (NMO210). Normal human serum (NHS) was run as a control. The blot is representative of three experiments.

We also analyzed the presence of FH-autoantibody immune complexes in the patients’ sera. To this end, IgG was precipitated using Protein G beads, and the bound proteins were eluted and analyzed by Western blot using the anti-FH mAb C18. FH was detected in the sera of patients #64, #84 and #210, displaying stronger bands compared with the IgG of a healthy individual, used as control (**Figure 3**). In addition, FHR-1 was detected in the case of patient #210, indicating cross-reactivity of the FH autoantibody with FHR-1 (**Figure 3**).

### Biological Features (Isotype, Titer, Avidity) of the FH Autoantibodies

Next, we determined the isotypes of the FH autoantibodies by ELISA. The FH autoantibodies were of the IgG3 isotype in all four patients, and had κ light chains, except for #210, who had λ light chains. The autoantibody titers were determined by applying serial serum dilutions on HSA- and FH-coated microtiter plate wells. The autoantibody titers of three of the patients were low (#64, 1:200; #84, 1:100, and #113, 1:200) and one (#210, 1:800) was higher, similar to a typical, autoimmune aHUS-associated high-titer anti-FH antibody, used as positive control.

**Figure 4:**
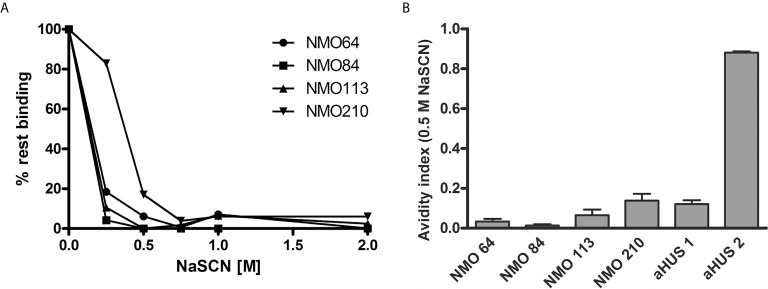
Avidity of FH autoantibodies. Avidity of the FH autoantibodies was determined by ELISA using NaSCN as a chaotropic agent. **(A)** Avidity profile of the autoantibodies. Residual binding of the FH autoantibodies from serum was measured after applying various NaSCN concentrations. Data are normalized to binding in the absence of NaSCN (=100% binding). A representative experiment out of two is shown. **(B)** Avidity indexes calculated at 0.5 M NaSCN. Avidity index is calculated as the ratio of remaining bound autoantibody after NaSCN elution (AU/ml)/autoantibody bound without NaSCN elution (AU/ml). Data are means ± SD of three measurements.

The avidity of the autoantibodies was determined using NaSCN to dissociate the FH-bound autoantibodies. A relatively low NaSCN concentration (0.5 M) was sufficient to dissociate the majority of the autoantibodies (**Figure 4A**). Autoantibodies of #210 showed slightly higher avidity, since ~80% of the autoantibodies were able to bind to FH at 0.25 M NaSCN, in contrast to the other patients’ autoantibodies. The small values of avidity indexes calculated at 0.5 M NaSCN (**Figure 4B**) indicated relatively low-avidity interaction between the autoantibodies of these NMOSD patients and FH.

### NMOSD-Associated FH Autoantibodies Bind to the FH C Terminus

Recombinant deletion mutants of FH, recombinant FHR-1 and, as a control, FHR-4B protein were used to determine the binding domains within FH. All four autoantibodies bound to the C-terminal domains of FH and cross-reacted with FHR-1 (**Table 2**). However, the binding profiles were slightly different: the three samples with low autoantibody titers bound strongly to FH15-20, FH19-20 and FHR-1, and comparatively weaker to purified, full-length FH, whereas the sample of patient #210, which showed a high background in ELISA and had relatively higher autoantibody titer, showed very weak binding to FH15-20, but bound equally well to FH19-20, FH and FHR-1 (**Table 2**).

**Table 2:** Summary of autoantibody binding sites on FH.

	NMO64	NMO84	NMO113	NMO210
FH	(+)	(+)	(+)	+
FH1-4	-	-	-	-
FH8-14	–	–	–	–
FH15-20	+	+	+	(+)
FH19-20	+	+	+	+
FHR-1	+	+	+	+
FHR-4B	–	–	–	–

The binding sites of the autoantibodies were determined using recombinant FH fragments containing domains 1-4 (FH1-4), 8-14 (FH8-14), 15-20 (FH15-20) and 19-20 (FH19-20), purified FH, the recombinant FH-related FHR-1 and FHR-4B proteins, the latter used as a negative control.

“(+)” indicates weak binding, “+” indicates prominent binding, “-” indicates no binding.

**Figure 5:**
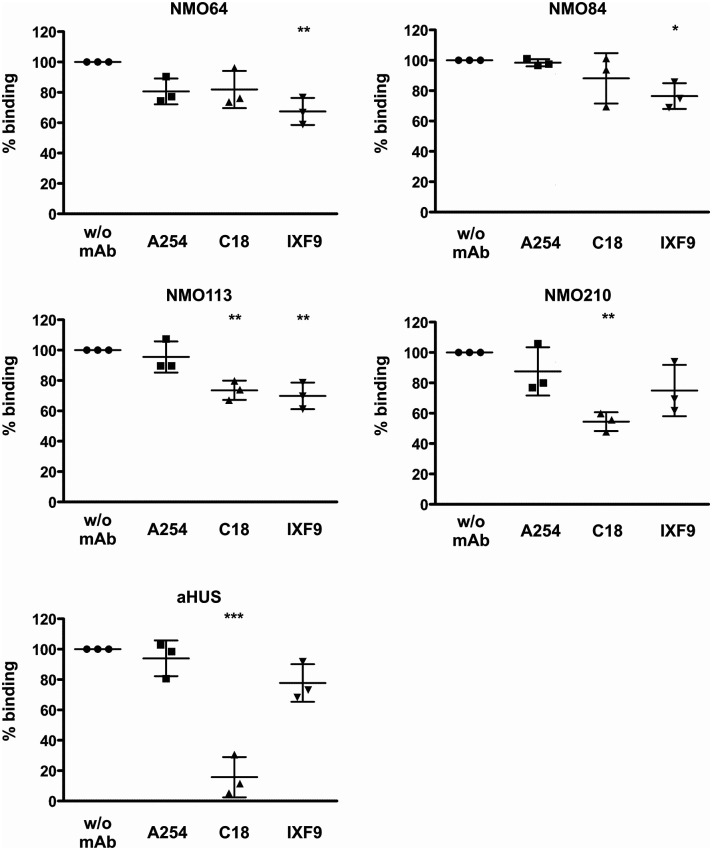
Inhibition of autoantibody binding to FH by mAbs. Immobilized FH was preincubated with the anti-FH mAbs A254 (binding in SCR1), C18 (binding in SCR20) and IXF9 (binding in SCR18-19), then serum samples of the FH autoantibody positive four NMOSD patients and of an aHUS patient, used as control, were added to the wells. Autoantibody binding was detected by HRP-conjugated anti-human IgG. Data are normalized to autoantibody binding in the absence of mAb. Data are mean ± SD of three measurements. *p < 0.05, **p < 0.01 and ***p < 0.001, one-way ANOVA.

To confirm and further characterize the binding site of these FH autoantibodies, two C-terminally binding mAbs against FH were used in competition assays. The inhibition profiles were heterogeneous. The mAb C18 recognizing an epitope in SCR20 (44) caused ~30% and ~50% inhibition of autoantibody binding to FH in the case of patients #113 and #210, respectively, whereas it had no significant inhibitory effect in the case of patients #64 and #84. The mAb IXF9 recognizing an epitope within FH SCR18-19 (41) inhibited autoantibody binding to FH by ~30% in the case of patients #64, #84 and #113, whereas its slight inhibitory effect did not reach statistical significance in the case of patient #210 (**Figure 5**). In the case of a control sample from an FH autoantibody positive aHUS patient, mAb IXF9 did not inhibit autoantibody binding to FH, but mAb C18 almost completely blocked autoantibody binding (**Figure 5**).

**Figure 6:**
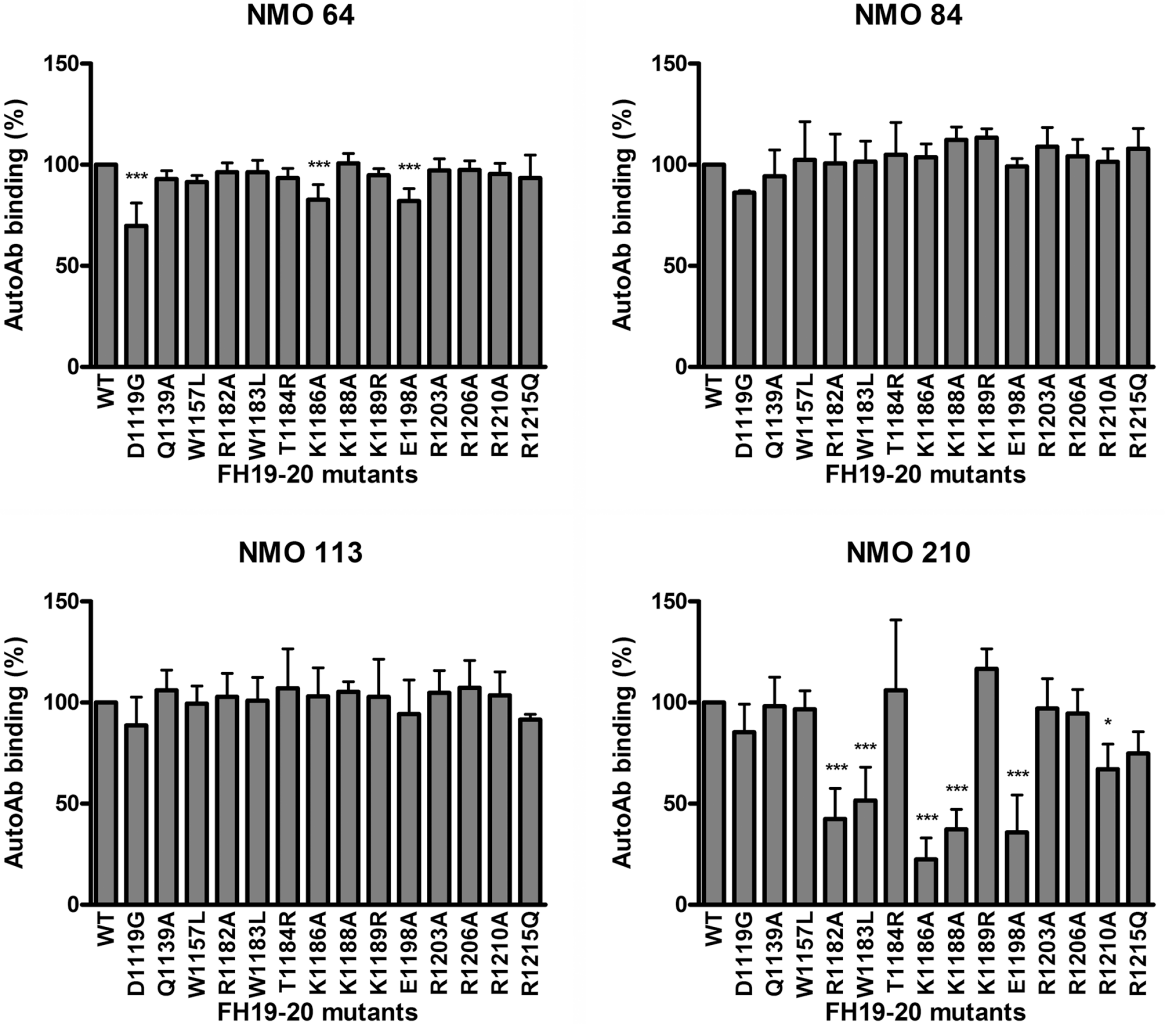
Epitope mapping using mutant FH19-20 fragments. The wild type FH19-20 fragment and 14 mutants containing single amino acid exchanges were immobilized in microtiter plate wells and incubated with patient serum. Autoantibody binding was detected using HRP-conjugated anti-human IgG. Data are mean ± SD of three experiments. *p < 0.05 and ***p < 0.001, one-way ANOVA.

To further analyze the autoantibody binding sites, 14 recombinant FH19-20 fragments with different single amino acid exchanges were used. With this approach, in the case of patient #210 strongly reduced autoantibody binding (50% or less binding) to the R1182A, W1183L, K1186A, K1188A and E1198A mutants was found, indicating that these residues are included in the binding site of the autoantibody (**Figure 6**). This site is within the hypervariable loop of FH SCR20 and coincides with the autoantibody epitope identified for most aHUS patients, as well as with the binding epitope of mAb C18 (44, 47, 54). Using the three other patients’ sera, no significant reduction in autoantibody binding to any of the tested mutants was found, except for ~25% or less inhibition of binding to the D1119G, K1186A and E1198A mutants in the case of #64, suggesting that their binding epitope lies elsewhere in SCR19 or SCR20.

**Figure 7:**
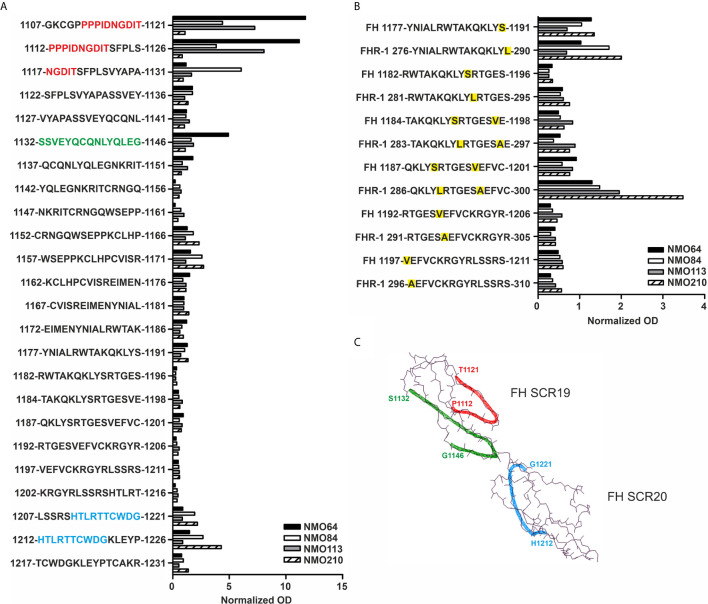
Linear epitope mapping of the FH autoantibodies. Overlapping 15-mer solid phase peptides **(A)** covering the 19-20 domains of FH and **(B)** containing the FH S1191L and V1197A FHR-1 specific amino acid exchanges (indicated by yellow highlighting) were incubated with patients’ sera. Autoantibody binding was detected using HRP-conjugated anti-human IgG, and is expressed as ratio of ODsample/ODmin, where ODsample is the mean of duplicate OD values of the patients’ samples, while ODmin represents the mean antibody binding to the negative control HSP480-489 peptide. On the *y* axis the initial and final amino acid of each tested peptide is displayed with the single-letter amino acid sequence indicated in between. **(C)** The schematic picture of the FH C-terminal domains shows the identified epitopes highlighted in red (1112-1121), green (1132-1146) and blue (1212-1221), corresponding to the color codes of the one-letter amino codes in A.

Linear epitope mapping of the autoantibodies was performed by peptide analysis using overlapping 15-mer peptides covering FH19-20. A heterogeneity of the binding sites of the autoantibodies was clearly detectable. Samples of #64 and #113 were positive for peptides derived from SCR19, the sample #210 reacted with peptides in SCR20, whereas autoantibodies of #84 bound to peptides of both SCRs (**Figure 7A**). Peptides corresponding to the differences in the FH SCR20-homolog domain of FHR-1, *i.e.* including the FH S1191L and V1197A amino acid exchanges, were also synthesized and analyzed. The peptide reactivity by the autoantibodies confirmed the cross-reactivity of the NMOSD-associated FH autoantibodies with FHR-1; interestingly, the sample of patient #210 showed strongly increased binding to the FHR-1 peptide 286-300 in comparison with the corresponding FH peptide 1187-1201 (**Figure 7B**). The identified peptides are shown on the FH19-20 structure in **Figure 7C**.

**Figure 8:**
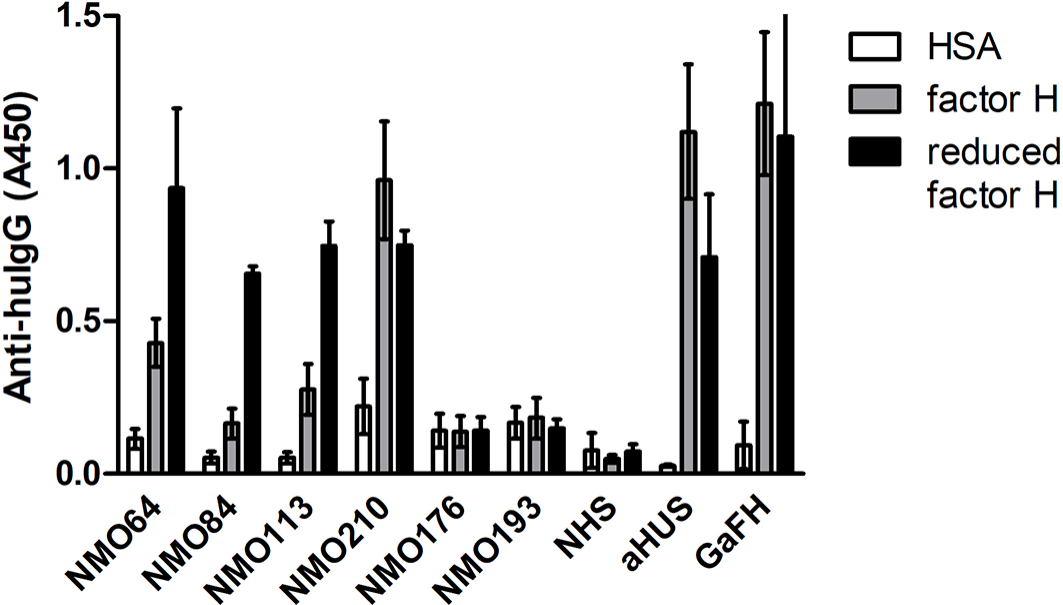
Autoantibody binding to reduced FH. Microplate wells were coated with equimolar amounts of HSA, FH and TCEP-treated, reduced FH, and after blocking, incubated with patients’ sera diluted 1:50 in PBS. Autoantibody binding was detected by HRP-conjugated anti-human IgG. As controls, sera of two FH autoantibody-negative NMO patients (#176 and #193), an aHUS patient and a healthy control (normal human serum, NHS) were used. Goat antiserum was used to prove that FH and reduced FH were immobilized on the plate. Data are mean ± SD of two experiments.

The SCR19 peptide 1114-PIDNGDIT-1121 was previously identified as a binding site for FH-autoantibodies detected in patients with non-small cell lung cancer, and the autoantibodies recognized FH particularly when FH was reduced. To further characterize the NMOSD-associated FH autoantibodies in this regard, the binding of autoantibodies to FH and TCEP-treated, reduced FH was compared. In this assay, the three samples that showed SCR19 reactivity in the epitope mapping assays, #64, #84 and #113 showed markedly increased binding to reduced FH, whereas in the case of #210 and an aHUS patient sample with known SCR20-binding autoantibodies, reduction of FH did not result in enhancement of reactivity (**Figure 8**).

### FH Autoantibodies of NMOSD Patients Inhibit the Interaction of FH With C3b

**Figure 9:**
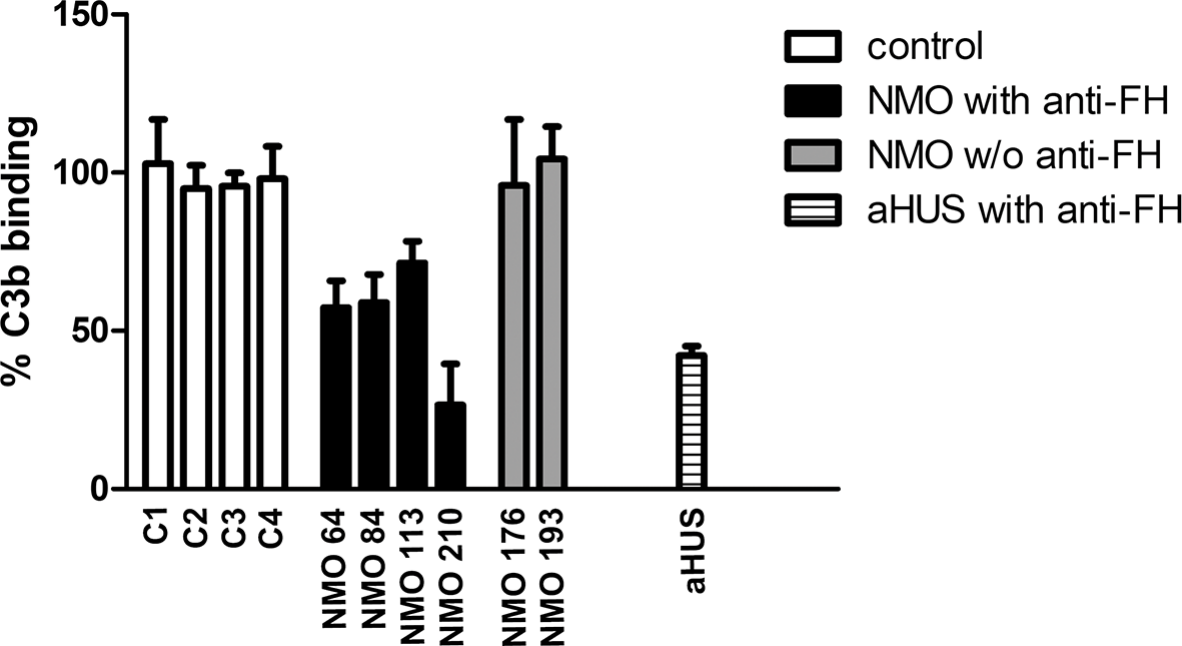
Inhibition of C3b binding to FH19-20 by FH autoantibodies. The immobilized FH19-20 fragment was incubated with IgG purified from the sera of NMOSD patients, healthy human sera (C) or serum of an aHUS patient with FH autoantibodies, then C3b was added. C3b binding was detected using HRP-conjugated anti-human C3 antibodies. Data are normalized to binding in the absence of IgG (=100%) and are means + SD of three measurements.

To assess whether FH autoantibodies of the NMOSD patients interfere with FH function, we analyzed the interaction of C3b with the FH19-20 fragment in the presence of the autoantibodies. IgG of patients #64 and #84 inhibited C3b binding to FH19-20 by ~40%, IgG of patient #113 by ~30%, and that of patient #210 by ~70%. By contrast, IgG derived from healthy individuals or NMOSD patients without autoantibodies to FH did not affect C3b binding (**Figure 9**).

## Discussion

Anti-complement autoantibodies are involved in several different diseases (12–14). For most autoantibodies a directly pathogenic role is not proven and therefore a matter of debate, such as in the case of C3NeF. FH autoantibodies appear pathogenic in aHUS and dense deposit disease, as functional consequence of the presence of the autoantibodies was described in terms of interfering with the interaction of FH with C3b and host cells and with the cofactor activity of FH, respectively (33, 34). FH autoantibodies are reported in 8-25% of aHUS patients in different cohorts and are strongly associated with homozygous deletion of the *CFHR1* gene, but are more rarely reported in patients with C3 glomerulopathies including dense deposit disease (32, 53, 55). On the other hand, FH autoantibodies might have a protective role such as described in patients with non-small cell lung cancer (46, 56). FH autoantibodies were also reported in inflammatory, autoimmune diseases where their role is less clear (57, 58). In addition, autoantibodies against the C3bBb convertase and its components C3b and FB are described in diseases associated with alternative complement pathway dysregulation (14, 45, 59–61). Therefore, we studied whether autoantibodies against C3bBb, C3b, FB and FH occur in NMOSD, a spectrum disease characterized by pathological complement activation.

FH autoantibodies were detected in four out of 45 NMOSD serum samples (~9%), while no antibodies against C3b, FB and C3bBb were found in these samples (**Figure 1**). The relevance of C1q autoantibodies that were detected in some samples (**Supplementary Figure 2**) should be investigated in the future. Three of the four identified FH autoantibodies were similar to each other by having low-titer, low-avidity autoantibodies and displaying identical binding profile to FH domains. The fourth autoantibody, that of patient #210, was clearly different and resembled more the aHUS-associated antibodies than the other three, by having high-titer FH autoantibodies and binding to the same hypervariable loop on SCR20 of FH (**Figures 5**–**9**) as the aHUS-associated autoantibodies (44). The autoantibodies bound to FH domains 19-20, and also recognized the homologous protein FHR-1, similar to FH autoantibodies associated with aHUS (44, 62, 63). However, in contrast to most autoantibody-positive aHUS patients, these four NMOSD patients did not lack the FHR-1 protein. The clear detectability of native FHR-1 in immune complexes of patient #210 could have been because of the higher autoantibody titer and avidity, as well as stronger reactivity with FHR-1 peptides in the case of this patient compared with the three other NMOSD patients (**Figures 3**, **4**, **7**). In both NMOSD and aHUS, the IgG3 isotype dominated among the FH autoantibodies, indicating infection- or inflammation-related generation of the autoreactive antibodies. Recently, based on the slight structural differences between the C termini of FH and FHR-1, and the lack of FHR-1 in most aHUS patients, we proposed a model for the generation of the aHUS-associated autoantibodies in the context of infection and induced neo-epitope due to slight structural change in the FH C terminus upon binding to microbial proteins (44). Collectively, the results of our experiments suggest a mechanism of autoantibody generation in the NMOSD patients different from that in the aHUS patients.

The FH19-20 and FHR-1 peptides recognized by the four NMOSD autoantibodies are in part similar to those described in aHUS patients (**Figure 7**) (54). These include the linear epitopes 1152-1171 in FH SCR19, which showed weak reactivity with the FH autoantibody positive NMOSD sera and strong reactivity with aHUS sera, and the peptides 1207-1226 in FH SCR20. The FH SCR19 peptides 1107-1131 and 1132-1146 showed reactivity only with the FH autoantibody positive NMOSD sera, but not with aHUS sera. The peptides FH SCR20 1177-1191 and the homologue FHR-1 276-290 showed only weak reactivity with the NMOSD sera compared with the strong reactivity of the autoantibody positive aHUS sera (**Figure 7**) (54). The FHR-1 peptide 286-300 showed strong reactivity with the serum of patient #210, and this peptide was non-reactive with aHUS sera. Interestingly, FH autoantibodies found in patients with non-small cell lung cancer recognize the peptide PIDNGDIT in FH SCR19, inhibit FH binding to lung carcinoma cells and cause increased C3-deposition when binding to FH that is already bound to the cancer cell surface (46). In our experiments, the linear epitope analysis showed the common recognition of epitope PPPIDNGDIT (SCR19 1107-1131) by FH autoantibodies of patients #64, #84 and #113, and these samples also showed enhanced reactivity with reduced FH (**Figures 7**-**8**). In the lung cancer study, the patients’ sera reacted strongly with reduced FH compared with the non-reduced protein, suggesting a cryptic epitope and/or a cancer-specific, posttranslational modification of the protein that is recognized by the autoantibodies. Similarly, it is possible that in NMOSD lesions the ongoing inflammation and damage of glial cells cause a slightly reducing microenvironment that may influence the conformation of FH, and allow for inflammation-driven induction of autoreactivity against this complement regulator.

Although recognizing different epitopes, autoantibodies of all patients affected binding of FH to C3b, with that of patient #210 strongly inhibiting the FH-C3b interaction (**Figure 9**). Since the interaction of the FH C terminus with C3b is critical for docking FH to C3b-covered surfaces and allowing FH to act as a regulator at the surface (37, 38, 64), the presence of these interfering autoantibodies may contribute to ongoing complement activation and damage of host cell surfaces, e.g. on astrocytes, where complement activation was initially triggered by NMO-IgG.

It is important to note that these NMOSD patients, particularly #210, despite having FH autoantibodies with overlapping characteristics and similar, C-terminal binding sites as the aHUS-associated FH autoantibodies, did not have manifest kidney disease. This might also be related to the relatively low avidity of the NMOSD-associated FH autoantibodies or difference in the exact binding site, compared to FH autoantibodies from aHUS patients’ sera. In addition, a fraction of the autoantibodies could bind FHR-1 instead of FH. However, it is theoretically possible that co-existence of AQP4-antibodies and FH-antibodies may contribute to subclinical impairment of kidney functions. Complement regulators are important in preventing peripheral organ injury in NMOSD patients and in the animal model of NMOSD (65, 66). However, the urine proteome and metabolome of NMOSD is different from multiple sclerosis (67, 68). Whether this reflects kidney alterations in a subgroup of patients with FH autoantibodies maybe worth investigating.

A characteristic feature of NMOSD is the increased frequency of associated autoantibodies and autoimmune diseases. Antibodies against gastrointestinal antigens may be present (69), and antinuclear antibodies were detected in 44% of patients with NMOSD (70). AQP4-antibodies were detected in patients with rheumatologic diseases in the presence of NMOSD-associated syndromes (71, 72), and temporal changes in SLE-associated antibody levels overlap with dynamics of AQP4-antibodies (73). Generation of autoantibodies against complement regulators, such as FH may be part of a co-existing condition in patients with susceptibility to multiple autoimmunity (72). We also describe that autoantibodies against the natural complement inhibitor FH in NMOSD patients impair the interaction of FH with C3b, which is the basis of its complement regulatory activity. This in turn may contribute to disease activity.

Limitations of our study include the low patient and sample number due to the rarity of the disease, and the lack of complement-active serial serum samples that restricted the breadth and the power of the analyses. At present, no clear conclusion on the correlation of the presence of FH autoantibodies with the clinical manifestation can be drawn. Analysis of additional patient cohorts and samples is expected to establish the frequency and the biological characteristics of the FH autoantibodies in NMOSD, and also whether and how these autoantibodies may contribute to the pathology of the disease and influence the clinical phenotype.

In conclusion, our results demonstrate that systemic FH autoantibodies are not uncommon in NMOSD, and they influence binding of FH to its main ligand, complement C3b. Our data also suggest that generation of autoantibodies against complement regulating factors among other autoantibodies may contribute to the complement-mediated damage in NMOSD.

## Author’s Note

Parts of this work were presented at the 25th International Complement Workshop, September 14-18, 2014, Rio de Janeiro, Brazil (*Mol. Immunol.* 2014, 61: 227).

## Data Availability Statement

The raw data supporting the conclusions of this article will be made available by the authors, without undue reservation.

## Ethics Statement

The studies involving human participants were reviewed and approved by National Ethical Committee (3893.316-12464/KK4/2010 and 42341-2/2013/EKU). The patients/participants provided their written informed consent to participate in this study.

## Author Contributions 

MJ initiated and supervised the study. BU and ZS performed autoantibody detection and characterization, Western blot and competition assays. KU designed and synthesized peptides. ET performed peptide binding assays. HN, ZI and ZP provided serum samples and patient data. SH and TJ provided recombinant mutant proteins. AE interpreted data. All authors contributed to the article and approved the submitted version. BU, ZI and MJ wrote the manuscript with the help of the other authors.

## Funding

This work was financially supported in part by the National Research, Development and Innovation Fund (OTKA, grant K 109055), by the Institutional Excellence Program to ELTE (NKFIH-1157/8/2019, D11206 to MJ), by the Hungarian Academy of Sciences (Lendület Program, grant LP2012-43 and research grant 01063 to MJ, and research grant TKI2017-02064 to. ZP), by Scleroseforeningen (R399-A28099-B15690 and R431-A29926 to ZI, and A31515 to HN), Lundbeckfonden (R118-A11472 to ZI), and Region of Southern Denmark (14/24200 to. ZI). The work also received support as part of FIEK_16 project; project no. FIEK_16-1-2016-0005 has been implemented with the support provided from the National Research, Development and Innovation Fund of Hungary, financed under the FIEK_16 funding scheme.

## Conflict of Interest

The authors declare that the research was conducted in the absence of any commercial or financial relationships that could be construed as a potential conflict of interest.

## Abbreviations

aHUS, atypical hemolytic uremic syndrome; AQP4, aquaporin 4; C3NeF, C3 nephritic factor; CNS, central nervous system; FB, factor B; FH, factor H; FHR-1, factor H-related protein 1; NMOSD, neuromyelitis optica spectrum disorder; SCR, short consensus repeat.
