# Supplementary material for: Autoantibodies Against the Complement Regulator Factor H in the Serum of Patients With Neuromyelitis Optica Spectrum Disorder
Source: Front Immunol. 2021 Apr 27;12:660382. doi: 10.3389/fimmu.2021.660382 (PMC8111293; doi:10.3389/fimmu.2021.660382)
Supplement: Supplementary file 1 [file DataSheet_1.pdf]

Supplementary Material

A

B

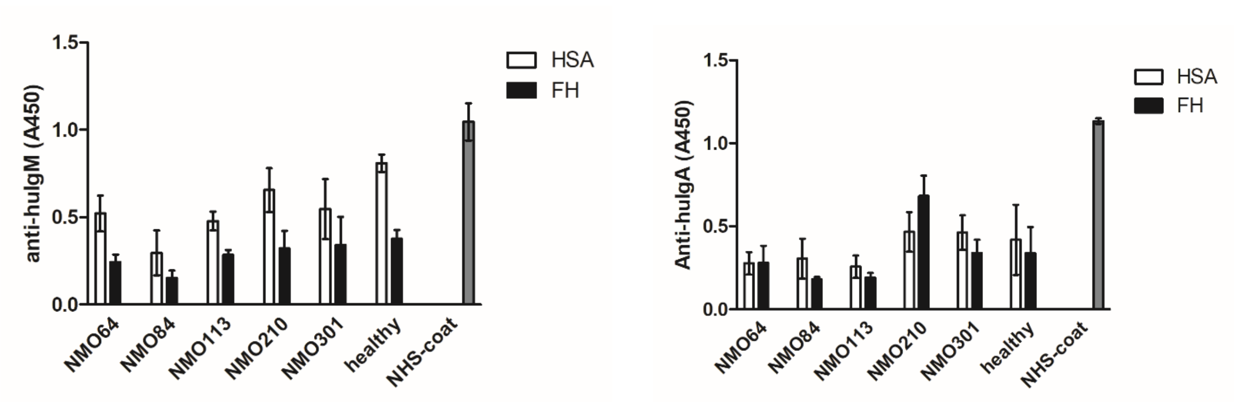

Supplementary Figure 1. IgM and IgA class FH autoantibodies in NMOSD patients. To assess the presence of other classes of FH autoantibodies, IgG was depleted from patients' sera using protein G beads. Depleted serum samples were added to microplate wells coated with HSA or FH and autoantibody binding was detected using HRP-conjugated anti-human IgM (A) or HRP-conjugated anti-human IgA (B). Normal human serum was immobilized as a technical control. Data are means of two experiments.

A

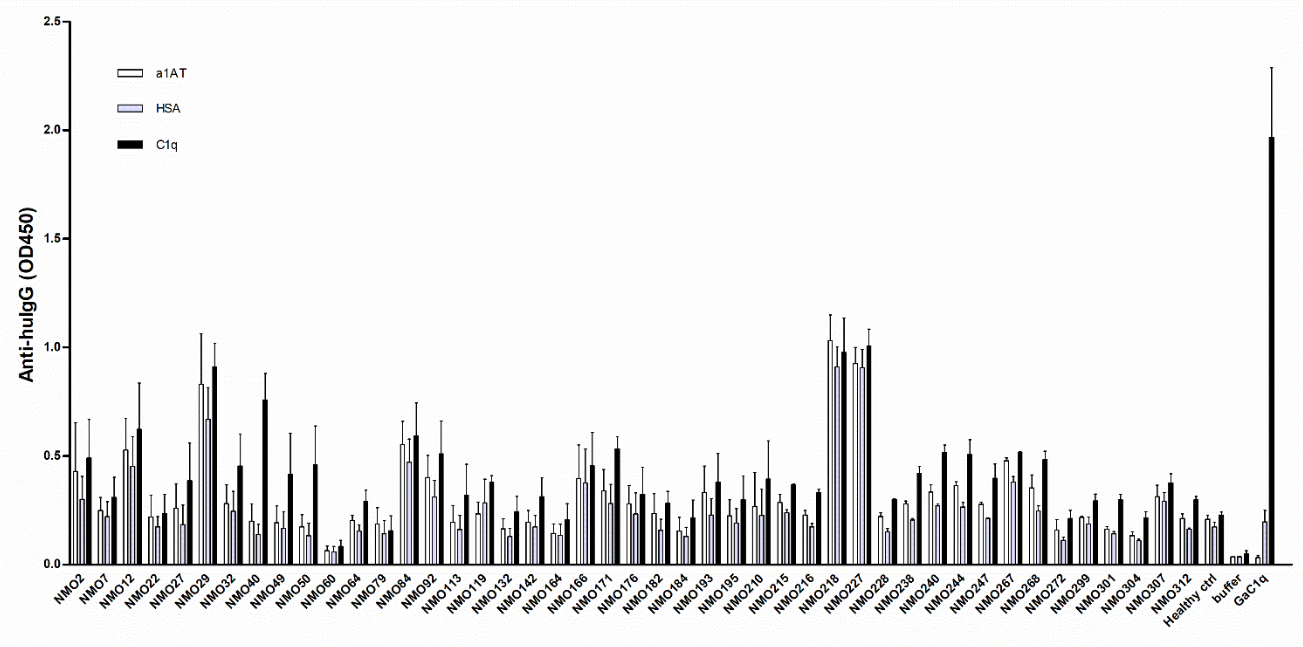

B

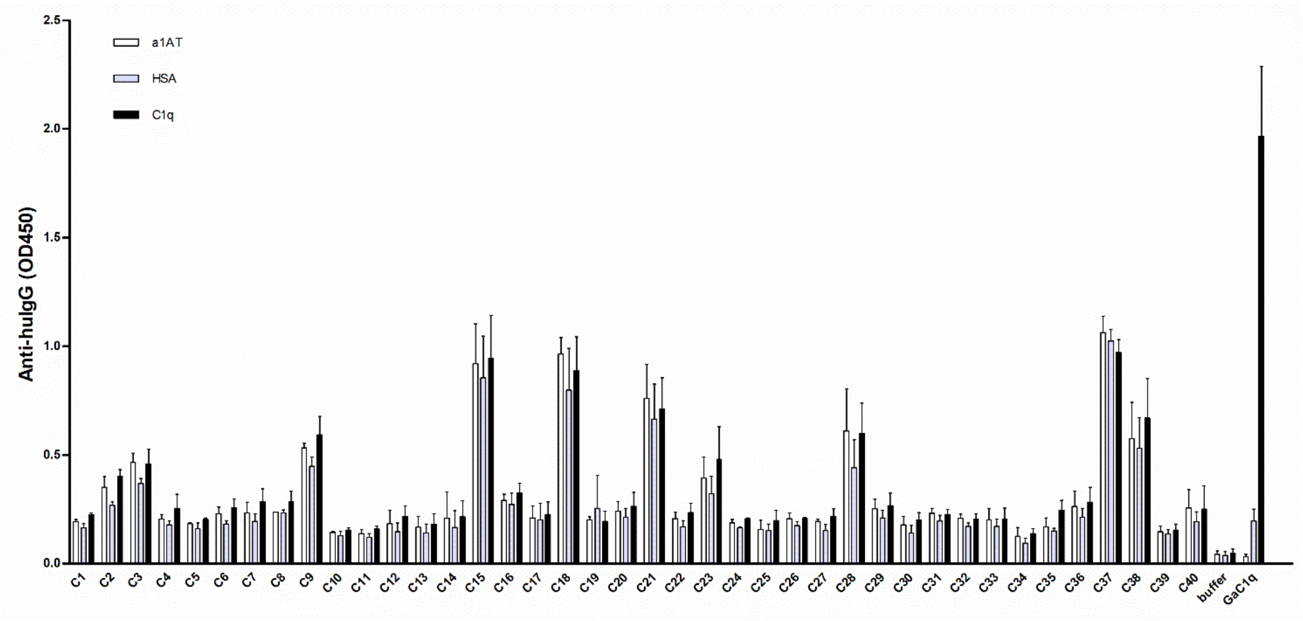

Supplementary Figure 2. Screening the NMOSD sera and healthy control samples for autoantibodies against C1q. Microplate wells were coated with C1q and, as negative controls, human serum albumin (HSA) and α1-antitrypsin (a1aT). After blocking, serum samples of (A) NMOSD patients and (B) that of healthy controls were added, and autoantibodies were detected by HRP-conjugated anti-human IgG. To exclude unspecific binding due to the "stickiness" of C1q, serum samples and the detecting antibodies were diluted in DPBS containing 1 M NaCl, which does not influence antigen-antibody binding. As a control, goat anti-C1q (GaC1q) and HRP-conjugated rabbit anti-goat Ig antibody were also used. Data are means ± SD of three experiments.
